# Mortality rate and cause among people with intellectual disabilities in South Korea: A nationwide representative cross-sectional study from 2015 to 2019

**DOI:** 10.1371/journal.pgph.0000744

**Published:** 2022-07-29

**Authors:** Ye-Soon Kim, Joo-Hee Kim, Sooyoung Kwon, Seunghee Ho

**Affiliations:** Department of Healthcare and Public Health Research, National Rehabilitation Center, Seoul, South Korea; Bangladesh University of Health Sciences, BANGLADESH

## Abstract

People with intellectual disabilities (ID) age faster and have a higher prevalence of degenerative diseases. The aim of this study was to identify the patterns/ causes of death among people with ID. We conducted a nationwide, retrospective, cross-sectional study of people with ID in South Korea. The database was compiled by merging data of people registered with ID, based on the 2015–2019 Standards from the Ministry of Health and Welfare, with the cause of death data published by the Korea National Statistical Office. The International Classification of Diseases-10 (ICD-10) was used to categorize causes of death. The mortality and standardized mortality rates were calculated and major causes of death were analyzed. As of 2019, the mortality rate of people with ID in South Korea was 784.6. The rate showed an irregular pattern, increasing or decreasing year by year (increasing from 725 in 2015 to 834 in 2018). The mortality rate of people with ID was approximately 1.4 times higher than the general population; the standardized mortality rate was 3.2 times higher. The main causes of death (48%) in people with ID were, in order of prevalence, circulatory diseases (cerebrovascular disease), neoplasms (malignant neoplasms of the digestive system), and diseases of the respiratory system (pneumonia). The leading causes of death (>60%) in the general population were, in order of prevalence, neoplasms, diseases of the circulatory system, and respiratory system diseases. An accurate understanding of the causes of death of people with ID is important to promote the development and application of health promotion programs and management thereof.

## Introduction

As of 2020, the number of people with intellectual disabilities (ID) in South Korea was 217,108 as registered by the South Korean government [1]. As with the general population, the average age at death of people with ID has increased in recent years. In South Korea, the average age at death of people with ID was 46.4 years in 2008. The average age at death increased by 0.8 years each year, and the average age reached 55.9 years in 2018 [2]. However, at the national level, worldwide, the mortality rate, death pattern, and cause of death in people with ID have not been actively studied [3]. There are only few studies on deaths of people with ID in South Korea. In addition, to the best of our knowledge, there have been no systematic reviews or meta-analyses of the causes of death in people with ID. A small number of related studies, identified through a search engine, were conducted on the death rate of deaf individuals or of all persons with disabilities and specific causes of death [4–8]. Previous studies on the mortality rate and cause of death of people with ID have assessed diverse target groups, study designs, and data analyses. The design of previous studies was of cross-sectional and cohort, and the study subjects were mainly the elderly age group [9–11]. Therefore, it is difficult to draw conclusions about the mortality rates and causes of death of people with ID, which are defined differently. People with ID are reported to age faster than the general population and they have more chronic and degenerative diseases [9–12]. It has also been reported that people with ID have a higher prevalence of mental health problems [13–15].

The Korean government has implemented a system to register and manage people with disabilities [16]. The South Korean government is probably one of the few countries with a system to register and manage all people diagnosed with ID. The system of “Bokjiro,” a welfare portal of the Ministry of Health and Welfare, includes persons with ID who receive services and support under the Welfare Act for Persons with Disabilities. The “Bokjiro” system contains personal information about people with ID. However, the death certificate of the National Statistical Office of Korea does not have a variable that can identify people with disabilities. A database of the causes of death of those with ID was established by linking information registered in the “Bokjiro” system, a population of people with ID in Korea, and death data from the National Statistical Office. The Korean government enacted the “Act on the Protection and Support of the Rights and Support of Persons with Developmental Disabilities Act”(Developmental Disabilities Act) in November 2015 and the “Act on the Guarantee of the Rights to Health and Medical Access for Persons with Disabilities Act” (Health Rights Act for Persons with Disabilities) in December 2017 [17, 18]. It is expected that the environment and life expectancy of people with ID will change due to the enactment of the Health Rights Act for Persons with Disabilities. Considering these positive social developments and changes in the living environments, it is estimated that there will be changes in the mortality rate and cause of death of people with ID in the future. In addition, it is desirable to know whether there is a difference in mortality rates between people with ID and the general population.

This study primarily aimed to determine the mortality rate of people with ID in comparison with that of the general population in South Korea. People with ID are expected to have higher mortality rates than the general population. The secondary objective was to provide internationally comparable standardized mortality data of people with ID in South Korea.

## Material and methods

### Data source

We obtained data on the people with a registered disabilities by the Ministry of Health and Welfare, from the end of the 2015–2019 period. In addition, data on the cause of death by the National Statistical Office were merged to establish a cause of death database for people with disabilities. A person with a registered disability is a person with a disability who, in accordance with Article 32 of the Welfare Act for the Disabled, or their legal representative or guardian prescribed by Presidential Decree, has registered their disability status and other matters prescribed by the Ordinance of the Ministry of Health and Welfare with the Special Self-Governing City Mayor, Special Self-Governing Province Governor, Si/Gun/Gu head [16]. The registration data for persons with disabilities include major variables such as gender, age, major type of disability, and comprehensive disability grade. The date, age, and cause of death are coded in the cause of death database for the disabled. The causes of death of the disabled were listed using the International Classification of Diseases-10 (ICD-10) codes. The cause of death information was recorded on the death certificate, and the World Health Organization (WHO) 103 classification criteria were used. This study used secondary data of deceased individuals. Therefore, it was practically impossible to obtain the consent of the research subjects during the course of the study, so the written consent of the research subjects was exempted. We study was approved by National Rehabilitation Institute Clinical Research Review Committee (NRC-2012-04-026).

### Statistical analysis

We presented descriptive statistics for all-cause mortality and mortality in people with ID. Mortality rate was calculated from the crude and age-adjusted mortality rates of people with ID in comparison with those of the general population in South Korea. The crude mortality rate is the ratio of the number of deaths during the year to the average population in that year. This value is expressed per 100,000 people. The age-adjusted mortality rate is the ratio of the number of deaths in a given age group to the population in that age group, expressed per 100,000 people. The age-adjusted mortality rate is expressed as standardized mortality rate (SMR). Data were analyzed using SAS 9.4 statistical software (SAS Institute Inc, Cary, NC).

## Results

### Demographic characteristics of people with intellectual disabilities and the general population

In South Korea, the mortality rate in people with ID was 794.9 in 2015 and 784.6 in 2019. The mortality rate for the general population was 541.5 in 2015 and 574.8 in 2019. The survey mortality rate was approximately 1.4 times higher for people with ID than for the general population. The mortality rate in males was high in both groups. Among the deaths of people with ID, those aged 0–59 years accounted for more than 50% of deaths of people with ID (61.98% in 2015 and 56.32% in 2019). However, in deaths of the general population, those aged 0–59 years accounted for less than 20% of deaths in the general population. Among the deaths of people with ID, deaths of those aged ≥60 years increased from approximately 38.02% in 2015 to 43.69% in 2019. Among the general population, deaths of those aged ≥60 years decreased from approximately 90.58% in 2015 to 83.48% in 2019 (Table 1). People with ID, especially the elderly, had an increased mortality rate. An aging population is believed to be one of the reasons for this finding.

**Table 1:** Demographic characteristics of deaths among people with intellectual disabilities and the general population in 2015 and 2019.

	Deaths among people with intellectual disability (ID)				Deaths among general population (GP)			
	2015		2019		2015		2019	
	n (%)	CMR	n (%)	CMR	n (%)	CMR	n (%)	CMR
Total	1,486	794.9	1,646	784.6	275,895	541.5	295,110	574.8
Sex								
Males	961 (64.67)	852.4	1,076 (65.37)	850.9	150,449 (54.53)	591.0	160,322 (54.33)	626.0
Females	525 (35.33)	707.5	570 (34.63)	683.9	125,446 (45.47)	492.1	134,788 (45.67)	523.9
Age group								
0–19 years	67 (4.51)	150.6	49 (2.98)	109.6	2,589 (0.94)	24.8	2,027 (0.69)	21.9
20–39 years	284 (19.11)	371.4	249 (15.13)	282.5	8,052 (2.92)	56.2	7,466 (2.53)	53.7
40–59 years	570 (38.36)	1,084.8	629 (38.21)	1,049.2	42,914 (15.55)	253.4	39,211 (13.29)	232.6
60–79 years	496 (33.38)	3,868.0	606 (36.82)	3,195.0	109,661 (39.75)	1,378.4	107,733 (36.51)	1,128.0
80+ years	69 (4.64)	11,201.3	113 (6.87)	10,700.8	112,638 (50.83)	8,584.7	138,602 (46.97)	7,833.7
Duration of disability								
< 10 years	414 (27.86)	532.0	266 (16.16)	401.3				
10–19 years	746 (50.20)	914.7	801 (48.66)	823.6				
20+ years	325 (21.87)	1,291.7	579 (35.18)	1,174.9				

n: number of deaths; CMR: crude mortality rate (per 100,000 people).

Table 2 shows the mortality rates for each group from 2015 to 2019. The mortality rate of people with ID showed an irregular pattern, increasing or decreasing year by year, increasing from 725 in 2015 to 834 in 2018. By contrast, the survey mortality rate of the general population increased from 541 in 2015 to 582 in 2018, but it decreased slightly in 2019. The mortality rate in people with ID was 1.4 times higher than that of the general population, while the SMR was 3.2 times higher than that of the general population.

**Table 2:** Mortality and mortality rates of people with intellectual disabilities and the general population from 2015 to 2019.

Years	Deaths among people with intellectual disability (ID)			Deaths among general population (GP)		
	N	CMR	SMR	N	CMR	SMR
2015	1486	794.9	1105.5	275895	541.5	347.6
2016	1578	820.1	1138.4	280827	549.4	337.2
2017	1436	725.2	988.2	285534	557.3	324.3
2018	1701	834.8	1080.2	298820	582.5	322.6
2019	1646	784.6	987.7	285110	574.8	305.4

N: number of deaths; CMR: crude mortality rate (per 100,000 people); SMR: standardized mortality rate (per 100,000 people).

### Causes of death in people with intellectual disabilities and the general population by year

Tables 3 and 4 present the number of deaths by cause, mortality rate, and SMR for people with ID and the general population. The main causes of death in people with ID were, in order of prevalence, circulatory diseases, neoplasms, and respiratory diseases. The main causes of death in the general population were, in order of prevalence, neoplasms, circulatory diseases, and respiratory diseases. These top three causes of death accounted for >60% of the total deaths in the general population. Among the major causes of death for people with ID, circulatory disease ranked first in the top five causes for the past five years, but the top second and third causes showed a change.

**Table 3:** Causes of death among people with intellectual disabilities based on ICD-10 codes from 2015 to 2019.

ICD-10 categories	2015 (N = 1,486)			2016 (N = 1,578)			2017 (N = 1,436)			2018 (N = 1,701)			2019 (N = 1,646)		
	n (%)	CSMR	SMR	n (%)	CSMR	SMR	n (%)	CSMR	SMR	n (%)	CSMR	SMR	n (%)	CSMR	SMR
Certain infectious and parasitic disease (A00-B99)	58 (3.90)	31.0	39.6	62 (3.93)	32.2	47.7	58 (4.04)	29.3	41.7	73 (4.29)	35.8	47.1	82 (4.98)	39.1	46.8
Neoplasms (C00-D48)	250 (16.82)	133.7	192.4	224 (14.20)	116.4	162.0	224 (15.60)	113.1	151.3	241 (14.17)	118.3	154.8	261 (15.86)	124.4	154.6
Diseases of the blood and blood-forming organs and certain disorders involving the immune mechanism (D50-D89)	13 (0.87)	7.0	8.9	4 (0.25)	2.1	3.1	7 (0.49)	3.5	5.3	9 (0.53)	4.4	5.1	6 (0.36)	2.9	3.3
Endocrine, nutritional and metabolic diseases (E00-E90)	45 (3.03)	24.1	32.1	66 (4.18)	34.3	47.2	57 (3.97)	28.8	32.8	83 (4.88)	40.7	48.5	55 (3.34)	26.2	30.3
Mental and behavioral disorders (F00-F99)	40 (2.69)	21.4	34.8	54 (3.42)	28.1	49.0	42 (2.92)	21.2	33.4	33 (1.94)	16.2	21.9	43 (2.61)	20.5	29.1
Diseases of the nervous system (G00-G99)	175 (11.78)	93.6	114.3	138 (8.75)	71.7	90.9	137 (9.54)	69.2	85.1	159 (9.35)	78.0	91.6	144 (8.75)	68.6	84.0
Diseases of the eye and adnexa (H00-H59)	0 (0.00)	NA	NA	0 (0.00)	NA	NA	0 (0.00)	NA	NA	0 (0.00)	NA	NA	0 (0.00)	NA	NA
Diseases of the ear and mastoid process (H60-H95)	0 (0.00)	NA	NA	0 (0.00)	NA	NA	0 (0.00)	NA	NA	0 (0.00)	NA	NA	0 (0.00)	NA	NA
Diseases of the circulatory system (I00-I99)	280 (18.84)	149.8	209.7	334 (21.17)	173.6	264.8	277 (19.29)	139.9	195.6	340 (19.99)	166.9	233.4	296 (17.98)	141.1	184.9
Diseases of the respiratory system (J00-J99)	188 (12.65)	100.6	167.3	220 (13.94)	114.3	166.3	215 (14.97)	108.6	159.3	279 (16.40)	136.9	197.1	262 (15.92)	124.9	173.4
Diseases of the digestive system (K00-K93)	94 (6.33)	50.3	66.1	89 (5.64)	46.3	54.2	88 (6.13)	44.4	54.3	91 (5.35)	44.7	48.1	91 (5.53)	43.4	52.2
Diseases of the skin and subcutaneous tissue (L00-L99)	5 (0.34)	2.7	2.7	8 (0.51)	4.2	3.9	3 (0.21)	1.5	2.4	8 (0.47)	3.9	6.3	8 (0.49)	3.8	4.3
Diseases of the musculoskeletal system and connective tissue (M00-M99)	9 (0.61)	4.8	5.8	5 (0.32)	2.6	3.1	5 (0.35)	2.5	2.6	7 (0.41)	3.4	4.3	5 (0.30)	2.4	2.0
Diseases of the genitourinary system (N00-N99)	32 (2.15)	17.1	26.3	24 (1.52)	12.5	20.1	32 (2.23)	16.2	24.2	37 (2.18)	18.2	25.5	37 (2.25)	17.6	29.4
Pregnancy, childbirth, and puerperium (O00-O99)	0 (0.00)	NA	NA	1 (0.06)	0.5	0.3	0 (0.00)	NA	NA	0 (0.00)	NA	NA	1 (0.06)	0.5	0.3
Certain conditions originating in the perinatal period (P00-P96)	0 (0.00)	NA	NA	0 (0.00)	NA	NA	0 (0.00)	NA	NA	0 (0.00)	NA	NA	0 (0.00)	NA	NA
Congenital malformations deformations and chromosomal abnormalities (Q00-Q99)	34 (2.29)	18.2	18.1	33 (2.09)	17.2	15.2	36 (2.51)	18.2	19.5	36 (2.12)	17.7	15.9	39 (2.37)	18.6	16.8
Symptoms, signs and abnormal clinical and laboratory findings, and NEC (R00-R99)	99 (6.66)	53.0	81.6	120 (7.60)	62.4	96.2	111 (7.73)	56.1	92.8	144 (8.47)	70.7	95.3	150 (9.11)	71.5	93.8
External causes of morbidity and mortality (V01-Y98)	164 (11.04)	87.7	106.0	196 (12.42)	101.9	114.5	144 (10.03)	72.7	87.8	161 (9.47)	79.0	85.4	166 (10.09)	79.1	82.4

n: number of deaths; CSMR: cause-specific mortality rate (per 100,000 people); SMR; standardized mortality rate (per 100,000 people); NA: not available.

**Table 4:** Causes of death among the general population based on ICD-10 codes from 2015 to 2019.

ICD-10 categories	2015 (N = 275,895)			2016 (N = 280,827)			2017 (N = 285,534)			2018 (N = 298,820)			2019 (N = 295,110)		
	n (%)	SMR	SMR	n (%)	CSMR	SMR	n (%)	CSMR	SMR	n (%)	CSMR	SMR	n (%)	CSMR	SMR
Certain infectious and parasitic disease (A00-B99)	7,295 (2.64)	14.3	8.8	7,946 (2.83)	15.5	9.0	7,986 (2.80)	15.6	8.5	8,746 (2.93)	17.0	8.8	8,692 (2.95)	16.9	8.2
Neoplasms (C00-D48)	78,281 (28.37)	153.6	102.9	79,729 (28.39)	156.0	100.2	80,320 (28.13)	156.8	96.1	80,747 (27.02)	157.4	92.1	82,844 (28.07)	161.4	90.7
Diseases of the blood and blood-forming organs and certain disorders involving the immune mechanism (D50-D89)	724 (0.26)	1.4	0.9	703 (0.25)	1.4	0.8	777 (0.27)	1.5	0.9	873 (0.29)	1.7	0.9	833 (0.28)	1.6	0.9
Endocrine, nutritional and metabolic diseases (E00-E90)	11,659 (4.23)	22.9	14.2	11,063 (3.94)	21.6	12.8	10,532 (3.69)	20.6	11.5	10,324 (3.45)	20.1	10.7	9,502 s(3.22)	18.5	9.5
Mental and behavioral disorders (F00-F99)	5,349 (1.94)	10.5	6.0	5,299 (1.89)	10.4	5.7	5,374 (1.88)	10.5	5.5	4,621 (1.55)	9.0	4.5	4,672 (1.58)	9.1	4.3
Diseases of the nervous system (G00-G99)	10,733 (3.89)	21.1	12.5	10,841 (3.86)	21.2	12.0	11,197 (3.92)	21.9	11.6	12,818 (4.29)	25.0	12.5	12,903 (4.37)	25.1	11.8
Diseases of the eye and adnexa (H00-H59)	0 (0.00)	NA	NA	1 (0.00)	0.0	0.0	0 (0.00)	NA	NA	5 (0.00)	0.0	0.0	1 (0.00)	0.0	0.0
Diseases of the ear and mastoid process (H60-H95)	3 (0.00)	0.0	0.0	4 (0.00)	0.0	0.0	6 (0.00)	0.0	0.0	9 (0.00)	0.0	0.0	6 (0.00)	0.0	0.0
Diseases of the circulatory system (I00-I99)	59,543 (21.58)	116.9	70.7	60,388 (21.50)	118.1	68.0	61,266 (21.46)	119.6	64.9	62,947 (21.07)	122.7	62.9	60,252 (20.42)	117.4	57.3
Diseases of the respiratory system (J00-J99)	27,806 (10.08)	54.6	31.1	29,404 (10.47)	57.5	31.2	32,644 (11.43)	63.7	32.2	37,763 (12.64)	73.6	34.9	36,655 (12.42)	71.4	31.9
Diseases of the digestive system (K00-K93)	11,709 (4.24)	23.0	15.7	11,949 (4.25)	23.4	15.3	12,132 (4.25)	23.7	14.9	12,401 (4.15)	24.2	14.6	11,963 (4.05)	23.3	13.6
Diseases of the skin and subcutaneous tissue (L00-L99)	470 (0.17)	0.9	0.5	518 (0.18)	1.0	0.6	620 (0.22)	1.2	0.6	612 (0.20)	1.2	0.6	617 (0.21)	1.2	0.5
Diseases of the musculoskeletal system and connective tissue (M00-M99)	1,491 (0.54)	2.9	1.8	1,496 (0.53)	2.9	1.7	1,521 (0.53)	3.0	1.6	1,412 (0.47)	2.8	1.5	1,366 (0.46)	2.7	1.4
Diseases of the genitourinary system (N00-N99)	6,546 (2.37)	12.8	7.8	7,168 (2.55)	14.0	8.0	7,582 (2.66)	14.8	7.8	8,160 (2.73)	15.9	7.9	8,565 (2.90)	16.7	7.8
Pregnancy, childbirth and puerperium (O00-O99)	42 (0.02)	0.1	0.1	37 (0.01)	0.1	0.1	32 (0.01)	0.1	0.1	41 (0.01)	0.1	0.1	36 (0.01)	0.1	0.1
Certain conditions originating in the perinatal period (P00-P96)	631 (0.23)	1.2	1.3	616 (0.22)	1.2	1.3	523 (0.18)	1.0	1.3	476 (0.16)	0.9	1.3	424 (0.14)	0.8	1.2
Congenital malformations deformations and chromosomal abnormalities (Q00-Q99)	433 (0.16)	0.8	0.9	426 (0.15)	0.8	0.9	371 (0.13)	0.7	0.8	359 (0.12)	0.7	0.8	321 (0.11)	0.6	0.7
Symptoms, signs and abnormal clinical and laboratory findings, and NEC (R00-R99)	24,396 (8.84)	47.9	27.7	25,021 (8.91)	49.0	26.8	25,497 (8.93)	49.8	25.8	28,466 (9.53)	55.5	27.6	28,176 (9.55)	54.9	26.1
External causes of morbidity and mortality (V01-Y98)	28,784 (10.43)	56.5	44.6	28,218 (10.05)	55.2	42.8	27,154 (9.51)	53.0	40.2	28,040 (9.38)	54.7	41.0	27,282 (9.24)	53.1	39.3

n: number of deaths; CSMR: cause-specific mortality rate (per 100,000 people); SMR; standardized mortality rate (per 100,000 people); NA: not available.

Most of the causes of death in people with ID had a higher mortality rate than that in the general population. However, in the case of neoplasms, the general population had a higher mortality rate than people with ID. The SMRs for all causes of death were higher in people with ID than in the general population. In addition, while the general population showed a certain pattern in which most causes of death increased or decreased, most of the causes of death in people with ID showed an irregular pattern. Among the causes of death in people with ID, only digestive diseases (K00-K93) showed a decreasing pattern of mortality.

Among the causes of death, congenital anomalies, deformities, and chromosomal abnormalities (Q00-Q99) showed the largest difference. The difference in mortality rates was more than 20 times between people with ID and the general population, followed by diseases of blood and hematopoietic organs and immune mechanisms. The next rankings were specific disorders (D50-D89); neurological diseases (G00-G99); skin and subcutaneous tissue diseases (L00-L99); and pregnancy, childbirth, and postpartum (O00-O99).

### Three leading causes of death within each elected major cause of death, based on ICD-10 categories among people with intellectual disabilities

Based on the ICD-10 codes, three major causes of death were additionally analyzed within each major cause of death in people with ID (Tables 5–7). Cerebrovascular disease (I60-I69) was the most common cause of death among people with ID who died due to diseases of the circulatory system. Other forms of heart disease (I30-I52) and ischemic heart disease (I20-I25) followed in prevalence. Conversely, acute rheumatic fever (I00-I02), chronic rheumatic heart disease (I05-I09), diseases of the veins, lymphatic vessels, and lymph nodes not elsewhere classified (I80-I89), and other unspecified disorders of the circulatory system (I95-I99) were infrequent. The mortality rates for ischemic heart disease (I20-I25), other forms of heart disease (I30-I52), and cerebrovascular disease (I60-I69) in 2015 were 27.3, 31.6, and 76.0, respectively. These diseases showed an irregular pattern of increasing or decreasing each year. Disease showed an irregular pattern when comparing 2015 and 2019, and the mortality rates in 2019 were 22.4, 39.6, and 62.9, respectively. There were decreased rates of cerebrovascular disease (I60-I69) and ischemic heart disease (I20-I25), but other forms of heart disease (I30-I52) showed an increasing pattern.

**Table 5:** Causes of death among people with intellectual disabilities by ICD-10-based diseases of the circulatory system from 2015 to 2019.

Causes of death	2015 (N = 280)			2016 (N = 334)			2017 (N = 277)			2018 (N = 340)			2019 (N = 296)		
	n (%)	CSMR	SMR	n (%)	CSMR	SMR	n (%)	CSMR	SMR	n (%)	CSMR	SMR	n (%)	CSMR	SMR
Acute rheumatic fever (I00-I02)	0 (0.00)	NA	NA	0 (0.00)	NA	NA	0 (0.00)	NA	NA	0 (0.00)	NA	NA	0 (0.00)	NA	NA
Chronic rheumatic heart diseases (I05-I09)	1 (0.36)	0.5	0.5	0 (0.00)	NA	NA	0 (0.00)	NA	NA	0 (0.00)	NA	NA	0 (0.00)	NA	NA
Hypertensive diseases (I10-I15)	17 (6.07)	9.1	15.0	21 (6.29)	10.9	22.2	12 (4.33)	6.1	8.5	24 (7.06)	11.8	22.9	18 (6.08)	8.6	15.2
Ischemic heart diseases (I20-I25)	51 (18.21)	27.3	35.6	65 (19.46)	33.8	46.7	56 (20.22)	28.3	38.4	69 (20.29)	33.9	44.4	47 (15.88)	22.4	28.2
Pulmonary heart disease and diseases of pulmonary circulation (I26-I28)	5 (1.79)	2.7	2.7	3 (0.90)	1.6	2.7	9 (3.25)	4.6	5.1	5 (1.47)	2.5	2.4	10 (3.38)	4.8	5.1
Other forms of heart disease (I30-I52)	59 (21.07)	31.6	37.0	90 (26.95)	46.8	65.0	79 (28.52)	39.9	51.8	95 (27.94)	46.6	57.1	83 (28.04)	39.6	50.1
Cerebrovascular diseases (I60-I69)	142 (50.71)	76.0	116.0	150 (44.91)	78.0	123.9	117 (42.24)	59.1	88.8	141 (41.47)	69.2	100.9	132 (44.59)	62.9	83.7
Diseases of arteries, arterioles and capillaries (I70-I79)	4 (1.43)	2.1	2.6	5 (1.50)	2.6	4.3	3 (1.08)	1.5	2.3	3 (0.88)	1.5	2.6	5 (1.69)	2.4	2.3
Diseases of veins, lymphatic vessels and lymph nodes, and NEC (I80-I89)	0 (0.00)	NA	NA	0 (0.00)	NA	NA	0 (0.00)	NA	NA	2 (0.59)	1.0	2.6	0 (0.00)	NA	NA
Other and unspecified disorders of the circulatory system (I95-I99)	1 (0.36)	0.5	0.3	0 (0.00)	NA	NA	1 (0.36)	0.5	0.9	1 (0.29)	0.5	0.5	1 (0.34)	0.5	0.4

n: number of deaths; CSMR: cause-specific mortality rate (per 100,000 people); SMR; age-adjusted standardized mortality rate (per 100,000 people); NA: Not Available.

**Table 6:** Death status of people with intellectual disabilities by ICD-10-based neoplasm diseases from 2015 to 2019.

ICD-10 categories	2015 (N = 250)			2016 (N = 224)			2017 (N = 224)			2018 (N = 241)			2019 (N = 261)		
	n (%)	CSMR	SMR	n (%)	CSMR	SMR	n (%)	CSMR	SMR	n (%)	CSMR	SMR	n (%)	CSMR	SMR
Malignant neoplasms of lip, oral cavity and pharynx (C00-C14)	3 (1.20)	1.6	1.4	4 (1.79)	2.1	2.9	2 (0.89)	1.0	2.5	6 (2.49)	3.0	3.7	13 (4.98)	6.2	7.5
Malignant neoplasms of digestive organs (C15-C26)	124 (49.60)	66.3	99.7	108 (48.21)	56.1	81.5	100 (44.64)	50.5	70.1	119 (49.38)	58.4	79.7	131 (50.19)	62.4	82.7
Malignant neoplasms of respiratory and intrathoracic organs (C30-C39)	39 (15.60)	20.9	36.8	26 (11.61)	13.5	23.5	26 (11.61)	13.1	19.2	38 (15.77)	18.7	25.6	42 (16.09)	20.0	27.0
Malignant neoplasms of bone and articular cartilage (C40-C41)	0 (0.00)	NA	NA	1 (0.45)	0.5	0.3	0 (0.00)	NA	NA	0 (0.00)	NA	NA	0 (0.00)	NA	NA
Malignant neoplasms of skin (C43-C44)	2 (0.80)	1.1	1.3	0 (0.00)	NA	NA	1 (0.45)	0.5	0.4	0 (0.00)	NA	NA	1 (0.38)	0.5	0.7
Malignant neoplasms of mesothelial and soft tissue (C45-C49)	2 (0.80)	1.1	3.4	3 (1.34)	1.6	1.1	6 (2.68)	3.0	3.6	2 (0.83)	1.0	1.0	4 (1.53)	1.9	2.2
Malignant neoplasms of breas, (C50)	9 (3.60)	4.8	6.1	18 (8.04)	9.4	10.1	20 (8.93)	10.1	10.1	13 (5.39)	6.4	6.9	10 (3.83)	4.8	4.3
Malignant neoplasms of female genital organs (C51-C58)	13 (5.20)	7.0	7.8	14 (6.25)	7.3	8.6	16 (7.14)	8.1	7.9	9 (3.73)	4.4	4.7	12 (4.60)	5.7	1.8
Malignant neoplasms of male genital organs (C60-C63)	3 (1.20)	1.6	2.3	2 (0.89))	1.0	1.4	1 (0.45)	0.5	1.0	5 (2.07)	2.5	4.4	0 (0.00)	NA	NA
Malignant neoplasms of urinary tract (C64-C68)	4 (1.60)	2.1	3.2	2 (0.89)	1.0	2.1	7 (3.13)	3.5	5.0	7 (2.90)	3.4	5.8	5 (1.92)	2.4	2.6
Malignant neoplasms of eye, brain and other parts of central nervous system (C69-C72)	17 (6.80)	9.1	9.8	11 (4.91)	5.7	5.5	8 (3.57)	4.0	3.8	12 (4.98)	5.9	5.4	7 (2.68)	3.3	3.3
Malignant neoplasms of thyroid and other endocrine glands (C73-C75)	2 (0.80)	1.1	1.3	2 (0.89)	1.0	1.7	4 (1.79)	2.0	2.0	1 (0.41)	0.5	0.4	2 (0.77)	1.0	0.9
Malignant neoplasms of ill-defined, secondary and unspecified sites (C76-C80)	6 (2.40)	3.2	4.3	4 (1.79)	2.1	5.7	9 (4.02)	4.6	8.4	4 (1.66)	2.0	2.9	3 (1.15)	1.4	1.7
Malignant neoplasms, stated or presumed to be primary, of lymphoid, hematopoietic and related tissue (C81-C96)	15 (6.00)	8.0	7.7	17 (7.59)	8.8	9.0	16 (7.14)	8.1	12.0	15 (6.22)	7.4	7.9	25 (9.58)	11.9	11.9
Malignant neoplasms of independent (primary) multiple sites (C97)	0 (0.00)	NA	NA	0 (0.00)	NA	NA	0 (0.00)	NA	NA	0 (0.00)	NA	NA	0 (0.00)	NA	NA
In situ neoplasms (D00-D09)	0 (0.00)	NA	NA	0 (0.00)	NA	NA	0 (0.00)	NA	NA	1 (0.41)	0.5	1.0	0 (0.00)	NA	NA
Benign neoplasms (D10-D36)	5 (2.00)	2.7	2.8	3 (1.34)	1.6	2.1	2 (0.89)	1.0	1.2	3 (1.24)	1.5	2.0	1 (0.38)	0.5	0.4
Neoplasms of uncertain or unknown behavior (D37-D48)	6 (2.40)	3.2	4.6	9 (4.02)	4.7	6.7	6 (2.68)	3.0	4.3	6 (2.49)	3.0	3.4	5 (1.92)	2.4	3.6

n: number of deaths; CSMR: cause-specific mortality rate (per 100,000 people); SMR; standardized mortality rate (per 100,000 people); NA: not available.

**Table 7:** Causes of death of people with intellectual disabilities by ICD-10-based diseases of the respiratory system from 2015 to 2019.

Causes of death	2015 (N = 188)			2016 (N = 220)			2017 (N = 215)			2018 (N = 279)			2019 (N = 262)		
	n (%)	CSMR	SMR	n (%)	CSMR	SMR	n (%)	CSMR	SMR	n (%)	CSMR	SMR	n (%)	CSMR	SMR
Acute upper respiratory infections (J00-J06)	0 (0.00)	NA	NA	0 (0.00)	NA	NA	0 (0.00)	NA	NA	1 (0.36)	0.5	0.8	1 (0.38)	0.5	0.7
Influenza and pneumonia (J09-J18)	134 (71.28)	71.7	115.2	154 (70.00)	80.0	115.5	151 (70.23)	76.3	108.9	209 (74.91)	102.6	144.2	205 (78.24)	97.7	135.2
Other acute lower respiratory infections (J20-J22)	0 (0.00)	NA	NA	1 (0.45)	0.5	0.5	2 (0.93)	1.0	2.0	0 (0.00)	NA	NA	0 (0.00)	NA	NA
Other diseases of upper respiratory tract (J30-J39)	0 (0.00)	NA	NA	1 (0.45)	0.5	0.5	0 (0.00)	NA	NA	0 (0.00)	NA	NA	0 (0.00)	NA	NA
Chronic lower respiratory diseases (J40-J47)	23 (12.23)	12.3	25.6	21 (9.55)	10.9	18.5	18 (8.37)	9.1	15.5	18 (6.45)	8.8	16.7	14 (5.34)	6.7	11.7
Lung diseases due to external agents (J60-J70)	19 (10.11)	10.2	14.8	22 (10.00)	11.4	17.7	28 (13.02)	14.1	22.5	36 (12.90)	17.7	23.9	21 (8.02)	10.0	13.1
Other respiratory diseases principally affecting the interstitium (J80-J84)	4 (2.13)	2.1	3.8	5 (2.27)	2.6	3.5	6 (2.79)	3.0	4.1	3 (1.08)	1.5	2.5	4 (1.53)	1.9	2.4
Suppurative and necrotic conditions of lower respiratory tract (J85-J86)	4 (2.13)	2.1	3.0	6 (2.73)	3.1	3.1	5 (2.33)	2.5	2.9	3 (1.08)	1.5	2.2	5 (1.91)	2.4	2.4
Other diseases of pleura (J90-J94)	2 (1.06)	1.1	2.6	1 (0.45)	0.5	0.4	1 (0.47)	0.5	0.5	2 (0.72)	1.0	1.3	2 (0.76)	1.0	1.1
Other diseases of the respiratory system (J95-J99)	2 (1.06)	1.1	2.3	9 (4.09)	4.7	6.6	4 (1.86)	2.0	3.0	7 (2.51)	3.4	5.5	10 (3.82)	4.8	6.8

n: number of deaths; CSMR: cause-specific mortality rate (per 100,000 people); SMR; age-adjusted standardized mortality rate (per 100,000 people); NA: Not Available.

In addition, neoplasms of the digestive organs (C15-C26) and respiratory and intrathoracic organs (C30-39) were common among people with ID who died due to neoplasia. This was followed by malignancies of the breast (C50), female reproductive system (C51-C58), eyes, brain and other parts of the central nervous system (C69-C72), lymph, malignant neoplasms, stated or presumed to be primary, of lymphoid, hematopoietic and related tissues (C81-96), etc. In addition, there were no deaths due to independent (primary) multi-site malignant neoplasms (C97). Neoplasms of the digestive tract (C15-C26) and respiratory and intrathoracic organs (C30-C39) were reported from 2015 to 2017. Conversely, breast neoplasms (C50) showed an increasing trend from 2015 to 2017 which then decreased from 2017 to 2019 (Table 6).

Influenza and pneumonia (J09-J18) were the most common diseases among people with ID who died due to respiratory system diseases. Next, our analysis showed that chronic lower respiratory disease (J40-J47) and lung disease caused by external factors (J60-J70) were the most frequent. Acute upper respiratory tract infection (J00-J06), other acute lower respiratory tract infections (J20-J22), and other diseases of the upper respiratory tract (J30-J39) were rarely found among the causes of death of people with ID. Influenza and pneumonia (J09-J18) accounted for more than 70% of the deaths of people with ID, showing an irregular pattern of increasing or decreasing year by year. Conversely, the mortality rate for chronic lower respiratory diseases (J40-J47) decreased every year from 12.3 in 2015 to 6.7 in 2019. The mortality rate of pulmonary diseases (J60-J70) caused by external factors increased every year until 2018, but declined in 2019 (Table 7).

## Discussion

This study considered the death patterns and specific causes of death of people with ID using the national database of causes of death for people with disabilities in South Korea. We investigated death rates and causes of death of people with ID who were registered with the national database from 2015 to 2019.

Our results revealed that the mortality rate of people with ID was approximately 1.4 times higher (SMR: 3.2 times higher) than that of the general population, and that the average age at death was low. Similar results were reported in the United States [19], Canada [20], United Kingdom [21], Australia [22], and Sweden [15, 23]. Males with ID had higher mortality and mortality rates than females [15, 20, 22, 23]. In the general population, the mortality rate in males was higher than that in females. Approximately 61.98% of the deaths of people with ID occurred in those aged <60 years, and the mortality rate was high among young people. This is consistent with the findings of Schoufour et al. [20] and Reppermund et al. [22].

Our study further examined the causes of death among people with ID in South Korea. The leading cause of death in people with ID was diseases of the circulatory system. Furthermore, circulatory system diseases among people with ID were more common than those among the general population. A similar observation was reported by a previous study, which revealed diseases of the circulatory system as the leading cause of death in people with ID [15]. In the current study, malignant neoplasm was the second most common cause of death in people with ID, followed by respiratory diseases. Studies by Patja et al. [24] (Finnish), Glover et al. [21] (United Kingdom), and Hirvikoski et al. [25] (Swedish) reported similar results on the common causes of death in people with ID.

Our study aimed to elucidate the major causes of death in people with ID. In addition, we compared the data of people with ID to those of the general population. The top three causes of death of people with ID in South Korea were circulatory system diseases, neoplasm, and diseases of the respiratory tract. A United Kingdom national population-based study found that adults with ID had the highest number of diseases in the order of circulatory system, respiratory system, neoplasm, nervous system, and digestive system [21]. In Sweden, the population with ID aged>55 years had the highest number of diseases in the order of circulatory system, respiratory system, neoplasia, nervous system, and mental and behavioral disorders [15]. According to data from the Australian cohort database for ID, the circulatory system, neoplasms, nervous system and sense organ disorders, and respiratory system rank highest [26]. In the Netherlands, population with ID aged>50 years had the highest number of diseases in the order of respiratory system, neoplasia, and circulatory system [27]. In addition, the South Korean population with ID had a higher mortality rate from all diseases than the general population. The rate was particularly high in patients with cerebrovascular diseases. Studies have shown that the population with ID is more vulnerable to cerebrovascular disease risk factors, morbidity, and mortality [28, 29].

This study used the data of people with ID who were registered in South Korea. Only few countries have a registry of persons with disabilities at the national level [15]. This study fills the gap in knowledge about the causes of death among people with ID. The main causes of death among people with ID could be more accurately identified by linking Korea’s data on registered persons with disabilities with data on the causes of death from the National Statistical Office. The death pattern of this population could be analyzed through the constructed ID death database, which could be helpful in health care planning for people with ID in the future. Considering the increasing evidence in this field, a systematic review or analysis of the pattern of causes of death in the life cycle of people with ID is necessary.

### Limitations

Although a database for the cause of death for people with ID in Korea was established, the database for the cause of death for the non-disabled could not be established. As such, data from the general population were used instead of the non-disabled; nence, it appears to be a database that includes people with ID. However, registered persons with disabilities, including those with ID, comprise approximately 5% of the Korean population. Finally, it can be said that there is no significant difference between the non-disabled death database and the general population death database. Therefore, the incidence and hazard risk of ID cannot be calculated owing to the characteristics of the data. Korea has a system in place for registering persons with ID. However, it is impossible to identify persons with ID who are not registered. In addition, children with ID may have been omitted from the registry. In the case of children, it is presumed that the lack of registration may be due to parents’ reluctance to register their children.

## Conclusions

Our study showed that the mortality rate for people with ID in South Korea was 1.4 times higher than that of the general population. In addition, our findings revealed that the average age at death of people with ID was low, meaning that these individuals had a shorter lifespan. The main causes of death in people with ID were circulatory disease (mainly cerebrovascular disease), neoplasm (digestive system neoplasm), and respiratory disease (mainly pneumonia). For the health management of people with ID, it is necessary to understand the cause of death statistics and to develop and apply customized health maintenance and management programs based on the data.
